# Additive Manufacturing of Drug-Eluting Multilayer Biodegradable Films

**DOI:** 10.3390/polym14204318

**Published:** 2022-10-14

**Authors:** Pavel I. Proshin, Arkady S. Abdurashitov, Olga A. Sindeeva, Anastasia A. Ivanova, Gleb B. Sukhorukov

**Affiliations:** 1 A.V. Zelmann Center for Neurobiology and Brain Rehabilitation, Skolkovo Institute of Science and Technology, Bolshoy Boulevard 30, 121205 Moscow, Russia; 2 Skoltech Center for Petroleum Science and Engineering, Skolkovo Institute of Science and Technology, Bolshoy Boulevard 30, 121205 Moscow, Russia; 3 School of Engineering and Materials Science, Queen Mary University of London, Mile End Road, London E1 4NS, UK; 4 Siberian State Medical University, Moskovskiy Trakt, 2, 634050 Tomsk, Russia

**Keywords:** biopolymers, drug-eluting coatings, zero-order release, 3D printing, polymer films, additive manufacturing

## Abstract

Drug-eluting films made of bioresorbable polymers are a widely used tool of modern personalized medicine. However, most currently existing methods of producing coatings do not go beyond the laboratory, as they have low encapsulation efficiency and/or difficulties in scaling up. The PLACE (Printed Layered Adjustable Cargo Encapsulation) technology proposed in this article uses an additive approach for film manufacturing. PLACE technology is accessible, scalable, and reproducible in any laboratory. As a demonstration of the technology capabilities, we fabricated layered drug-eluting polyglycolic acid films containing different concentrations of Cefazolin antibiotic. The influence of the amount of loaded drug component on the film production process and the release kinetics was studied. The specific loading of drugs was significantly increased to 200–400 µg/cm^2^ while maintaining the uniform release of Cefazolin antibiotic in a dosage sufficient for local antimicrobial therapy for 14 days. The fact that the further increase in the drug amount results in the crystallization of a substance, which can lead to specific defects in the cover film formation and accelerated one-week cargo release, was also shown, and options for further technology development were proposed.

## 1. Introduction

Site-specific drug delivery by using bioresorbable polymer drug-eluting films (DEFs) has been used already over two decades and has become widespread in applications of various medical devices [1,2]. The drug coating made of various biocompatible polymers, presumably polylactic acid (PLA), polycaprolactone (PCL), polyglycolic acid (PGA) and their copolymers, maintains a drug concentration at the application site that is similar or even superior to that of systemic therapy, while using a much lower total dose [3]. Reducing systemic toxicity is consistent with the principles of personalized medicine, as it increases the variability in the choice of drugs for patients with intolerance to high systemic drug concentrations and the effectiveness of treatment. Because of that, the use of antibiotic-releasing antimicrobial films significantly reduces the risks of bacterial contamination of dental devices and orthopedic implants [4,5,6], and the use of a combination of a drug and a coronary stent in the form of a drug-eluting stent has established itself as the most popular treatment option for restoring blood flow in occluded vessels [7].

The two most common approaches in DEFs manufacturing are: (i) a composite film forming, where drug powder is simply mixed with a coating polymer layer; and (ii) template methods, where a target drug is deposited into special isolated ordered microcontainers formed onto coating film or directly on a medical device surface using special templates [8,9,10]. The formation of microcavities and containers on the surface of the implantable devices is a convenient method to reduce the number of steps in the production scheme. At the same time, this approach cannot be called universal, since the product must be initially designed taking into account the surface roughness and the strength characteristics of porous materials [11,12]. In this paper, we observe methods that allow us to functionalize an already finished product such as the application of composite films and micro-container films.

Composite methods are simple and make it possible to produce DEFs of large area in industrial scale and allow obtaining multilayer coatings capable of releasing different drugs in different time intervals. The downside of these film fabrication methods is their ineffectiveness when used for long-term prophylaxis and therapy, which is largely due to the low load capacity of composite layers due to volumetric erosion risk and insufficient drug release time [13]. On the contrary, template methods are capable of retaining a significant amount of substance with various molecular weights. Over the past few years, the effectiveness of using such coatings for the encapsulation of low molecular weight drugs and dyes has been shown, and also, in certain modifications, the triggered release of the load under directed exposure to ultrasonic, magnetic or laser radiation has been demonstrated [8,14,15,16,17]. The sticking point of this approach is the complexity of the manufacturing system transferred from laboratory technique to real production. As in practical use, having a template process limits manufacturing flexibility, as every new product type will always require a new template to be made. Semi-manual lab methods for applying films and loading drugs further complicate the scaling up of the manufacturing process of template-based DEFs and therefore hinder the use of films in real life.

In this work, we move away from the micron size and using additive technologies, as we explore combining the performance of template-based films with the processability of the composite film’s approach. We have obtained easy to organize in the laboratory and simply scalable to industrial level flexible technology named “PLACE”: Printed Layered Adjustable Cargo Encapsulation. The proposed approach allows producing the large-area DEFs with advanced features, namely the precisely controlled loading of drug with significant payload up to 1 mg/cm^2^, and the possibility of manufacturing multilayered films by altering the different drugs and biopolymers within layers, thus obtaining programmable multifunctional coatings. As a test of technology capabilities, we fabricated layered drug-eluting polyglycolic acid films containing Cefazolin antibiotic agent, which is suitable for prosthesis surface modification, and use as antibacterial dressing. In this study, we investigated how the amount of loaded material affects the film production process and the release kinetics.

## 2. Materials and Methods

### 2.1. Chemical Materials

Polyglycolic-*co*-lactic acid (PLGA) granules PURASORB^®^ PDLG 5010 were obtained from Corbion N.V. (Amsterdam, The Netherlands). Acetone, ethyl acetate and sodium chloride were obtained from Sigma-Aldrich (Darmstadt, Germany), polyvinyl alcohol (PVA) powder with the molecular weight (MW) 72,000 g/mol and with the degree of hydrolysis 85–89% were supplied from AppliChem GmbH (Darmstadt, Germany); all reagents were used as received. For the solutions preparation, the deionized (DI) water (electric conductivity of about 18.2 MΩ·m^−1^ at 25 °C) prepared by the Milli-Q Plus185 from Millipore (Darmstadt, Germany) water purification system was used. Cefazolin sodium salt (Cefazolin) was obtained from the pharmaceutical company LEKKO (Volginsky, Russia). Novaprint PP-GF polypropylene filament (Moscow, Russia) was used to print upgrade parts.

### 2.2. Multilayer Drug-Eluting Films Preparation

**Figure 1:**
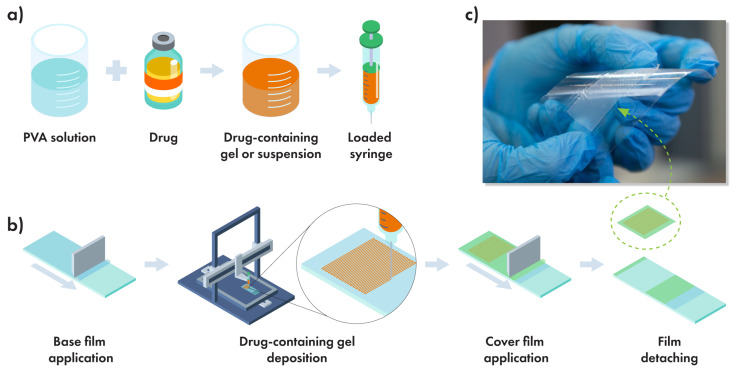
The design of the PLACE approach. (**a**) Mixing of the drug-containing matrix, (**b**) film fabrication pathway, and freestanding ready-to-use film (**c**).

DEFs were prepared by deposition of the PVA-contained drug matrix (Figure 1a) onto PLGA precoated polypropylene (PP) substrate using an upgraded 3D printer machine with a syringe extruder (Figure 1b). After drying, the multilayer film was detached from the PP substrate for further usage. The release properties of the films were studied without removing them from the substrate.

#### 2.2.1. Polymer Film Application

Polymer films were prepared using the Dr. Blade application technique. Dr. Blade coating is a reliable and scalable method for obtaining homogeneous polymer films with a minimum of solution losses [18,19]. The technique consists in rolling the polymer solution over the substrate with a special applicator blade moving along the surface at a fixed distance and creating a wet film. The 10 wt % solution of PLGA in acetone:ethyl acetate mixture (2:8) was used. Polypropylene substrates were fixed on a vacuum perforated table. Base 3 µm PLGA films were blade coated in ambient conditions with a coating speed (CS) of 20 mm/s, and a Baker applicator gap (AG, the gap between the meniscus guide and the substrate) of 100 µm, solution aliquote 150 µL. The coated substrates were dried at 50 °C for 10 min, after which the PVA matrix was deposited. Cover 10 µm films were prepared the same way as base films but using a larger AG of 400 µm and the PLGA solution aliquote of 800 µL.

#### 2.2.2. Drug Matrix Preparation

All PVA solutions were prepared at the magnetic stirrer by dissolving the polymer in the DI water for 2 h at 90 °C. The obtained base solutions were stored in tightly sealed vials in the refrigerator. To prepare three sets of samples—**Cef100**, **Cef200** and **Cef400**—100, 200 and 400 mg of Cefazolin sodium salt were dissolved at ambient temperature in 1000 µL of 9% PVA solution, respectively. The obtained solutions were centrifuged at 10,000 rpm for 1 min to remove the air bubbles.

#### 2.2.3. Drug Deposition, Basic Print Settings

A PVA matrix was deposited using an upgraded 3D printer machine on the PP substrate with precoated PLGA films in ambient conditions with a 23 G needle (ID 300 µm), Z-offset (ZO, the gap between the needle point and substrate) of 150 µm, gel flow (GF) rate of 850 µL/h, printhead linear speed (LS) of 20 mm/s, and acceleration of 5000 mm/s^2^. The coated substrates were dried at 40 °C for 10 min in the vacuum oven.

### 2.3. Three-Dimensional (3D) Printing Software and Hardware

All 3D models for the 3D printer upgrade were prepared in the Fusion360 CAD program (Autodesk) and printed using a UNI 3D printer. The sample models we wanted to print were prepared in the Fusion360 CAD program as solid blocks with dimensions of 10 × 10 × 0.01 mm and saved as an *.stl* file. Obtained models were sliced in an IdeaMaker slicer program (Raise3D) with the following parameters: extrusion width = 0.3 mm; bottom and top solid fill layers = 0; infill density = 50%; printing speed = 20 mm/s; travel speed = 100 mm/s, “Rectilinear” infill pattern type. The obtained g-code was used without any modifications.

### 2.4. DEFs Sample Characterization

#### 2.4.1. Polymer Film Thickness Investigation

The thickness of the polymer films was calculated from the analysis of SEM images in ImageJ software. The measurements were carried out for images of films cryosections at several points. The accuracy of the method was 0.1 micron for images with a 2500× magnification.

#### 2.4.2. Prolonged Drug Release Investigation

**Figure A1:**
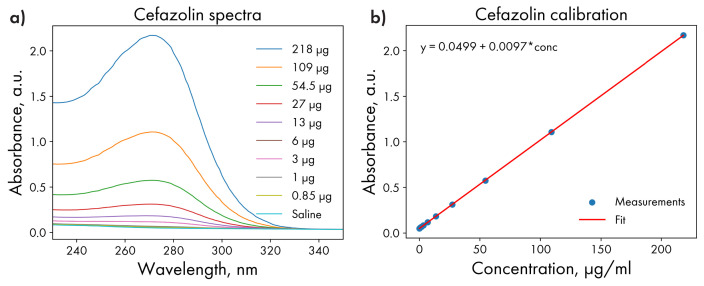
Absorbtion spectra (**a**) and calibration plots (**b**) for Cefazolin.

Cefazolin sodium salt is a highly water-soluble antibiotic agent. Concentration measurements were performed by the absorption spectra analysis in the NUV region by a characteristic absorption band at 271 nm [20]. A set of absorption spectra of the antibiotic solution in saline (0.9 wt % NaCl) was obtained in the concentration range from 0.8 to 200 µg/mL (Figure A1a). According to the obtained data, a calibration curve was plotted (Figure A1b), which was used to calculate the Cefazolin content in the samples during the experiment. For prolonged release of drug from films, the prepared films were placed in saline and incubated in a thermoshaker at 37 °C with constant stirring of 300 rpm for 14 days. The samples were transferred to clean saline daily.

### 2.5. Characterization Technique

Scanning electron microscopy (SEM) measurements were performed with a VEGA III (TESCAN, Brno, Czech Republic) microscope at an operating voltage of 5 kV. Before measurement, gold was deposited onto the sample (5 nm gold layer) using an Emitech K350 sputter-coater (Quorum Technologies Ltd., Ashford, UK). Absorption spectra were recorded using a Tecan Infinite 200Pro Microplate reader (Tecan Trading AG, Männedorf, Switzerland). Edge samples for SEM were prepared using a Leica CM1950 cryostat (Leica Biosystems Nussloch GmbH, Nußloch, Germany). Viscosity measurements were performed with Anton Paar MCR302 rheometer (Anton Paar GmbH, Graz, Austria).

## 3. Results and Discussion

### 3.1. Strategy Development

**Figure 2:**
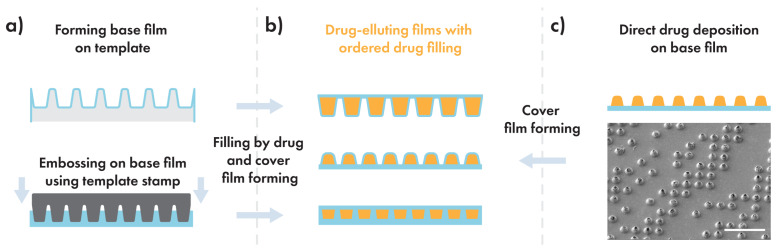
Simplified process of DEFs production. (**a**) Template-based methods, (**b**) DEFs represented as array of microcontainers filled with drugs and sealed between two polymer films, (**c**) direct drug deposition approach and SEM image of drug deposited onto flat PLA film as separate piles using the PDMS stamp-assisted transfer method; scale bar 100 µm [21].

The classical template-based DEFs represent an array of microcontainers filled with drugs and sealed between two polymer films and can be considered in the context of three main steps: formation of the containers on the base polymer film, loading of the medicinal substance, and application of the cover film. In the first step, a base polymer film is usually formed on a stamp made of polydimethylsiloxane (PDMS) by the dip-coating method or shaped by the embossing method using silicon or nickel matrices (Figure 2a). Next, the drug is loaded into the isolated containers in the form of a dry powder, and then, the filled containers are sealed by a cover polymer film (Figure 2b).

The first two steps represented the most challenging and innovative procedures. Indeed, the usual size of microchamber structures does not exceed 100 microns. Therefore, all templates must be manufactured with high precision, since any deviation in the dimensions and flatness of its sides will directly affect the quality of the formed surface. For example, good results were achieved when working with small PDMS stamps of 1–5 cm^2^, while using stamps with an area of more than 8 cm^2^ resulted in many defects caused by the curvature of the stamp surface as well as significant stamp bending due to swelling in organic solvents [22]. Additionally, an increase in the template area leads to an increase in the cost of its manufacture, whether it is a lithographic matrix or direct pyrolysis of PDMS [23]. Powder loading is also a sticking point for scaling up the DEFs manufacturing process. This process is difficult to automate, since the medicinal powders differ greatly in their properties such as granule size, hygroscopicity, electrification, and compressibility; all of these directly affect the loading efficiency. Thus, the loading step remains subject to individual operator skills. It should be pointed out that many drugs are quite expensive, and many application methods, such as spraying or screen printing, generate a large amount of hazardous waste. Thus, the lack of methods to produce large-area films with good repeatability significantly hinders the development of the idea of using DEFs in medicine.

The biopolymers used in the industry acts as dense membrane only permeable to low molecular weight gases and water vapors due to the sorption–desorption process [24]. Thus, theoretically, these films should hold the entire volume of the loaded drug before degradation begins, being in essence an example of first-order delivery systems [25]. In practice, the release of the substance from the coating begins much earlier due to stochastically occurring defects in film fabrication, i.e., various micropores and cracks caused by the roughness of the template and loading, contamination, air bubbles in the solution, and physical impact on the thin and brittle polymer film. The actual kinetics will depend on the magnitude and number of these defects, which for water-soluble low-molecular-weight drugs is mainly controlled by the diffusion process [25,26]. Thus, to achieve an ideal monotonic zero-order release, it is essential to first maximize the retention of a large portion of the drug by minimizing the defectiveness of the film and then provide the necessary level of “porosity” of the film to allow the drug to exit.

A promising way to overcome this problem can be the rejection of the use of templates and the simplification of the DEF fabrication process. Thus, the drug layer should be applied precisely on the flat base film by a printing method, in which the powder will be dissolved in water or bound by a binder—in this way, we can get rid of film damage due to interaction with the template. The next step is to raise the mechanical strength of the covering film—just increase its thickness to resist defects caused by physical manipulations. We have studied several available manual methods to transfer drugs onto flat film such as transfer using a PDMS template (SEM image in Figure 2c), but the simplest and seemingly the most interesting method was 3D printing using a CNC-controlled 3D printer, such as those used for 3D bioprinting. This printing method is very flexible and allows quickly forming coatings of a large area and a predetermined shape without any hard physical impact on the film. Furthermore, on the laboratory scale of a production, there is no need to purchase complex expensive equipment, since a regular 3D printer with a minimal upgrade can be utilized.

Thus, the final concept of the new production approach represents three sequential steps: base film forming using the Dr. Blade technique—a robust method to obtain a thick and uniform polymer films, printing the needed pattern using a 3D printer, and the last step—sealing with cover polymer layer using Dr. Blade again. Moreover, the second and third steps can alternate several times to obtain multilayered coatings.

### 3.2. Printing Part

**Figure 3:**
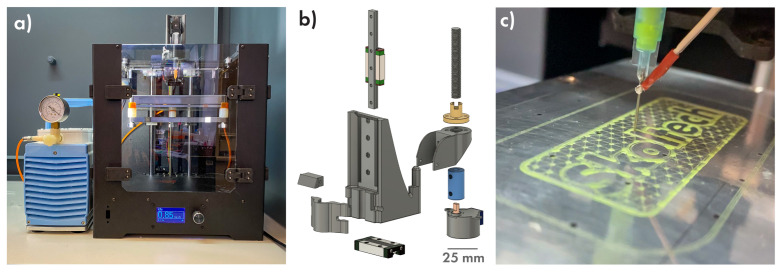
(**a**) Commercial 3D printer upgraded to use for drug gel printing, syringe extruder 3D model (**b**) and a complex shape print example (**c**).

The core component of the proposed technology is a Computer Numerical Controlled machine that dispenses drug-contained gel over the base biopolymer film in a programmable pattern. It can be constructed on the base of any commercial 3D printer using a minimal number of additional parts. In this study, we used a 3D printer kit from the local manufacturer “VS3D Printers” (Figure 3a). It was decided to use the direct mechanical syringe pump, where a syringe is fastened directly on the printhead and the pressure on the piston is controlled by the stepper motor, because such a kind of pump is easy to assemble and has a low inertia level. The pumping motor is controlled by an Arduino Nano board and ran separately from a 3D printer; thus, there is no need to modify the machine’s firmware. The resulted syringe pump extruder (Figure 3b) is suitable for use in any printer with the axis mechanics based on MGN9 linear rails. Links to a repository with all 3D models, circuit diagrams, and Arduino sketches can be found in the Supplementary Materials. The pump was assembled, calibrated and integrated into the 3D printer. Since in this study, we used our coatings to change the surface of the bone implants and dressing material, we needed to cover quite large areas and ensure a high drug load. In this regard, the needle 23 G (330 µm ID) gauge was chosen as it can provide a sufficiently narrow extrusion width and at the same time allow working with an effective flow. To supply a proper printing regime and prevent the accumulation of excess material on the needle point, a Z-offset equal to the diameter of the needle was used.

The use of a CNC pump made it possible to use various drug application patterns from the simplest (clusters, nets, tracks, and combined variants) to complex images (Figure 3c). As an infill pattern for DEFs, the 50% infill snake-like variant was suggested as the simplest and fastest type of continuous filling without crossing lines.

The next step dealt with the drug loading stage, which is the most important and the most complicated part of the manufacturing process. As a basis, several strict requirements were formulated: the medium that binds drug particles should be water-based and should not contain harmful and non-biocompatible components, have low reactivity, and, finally, be viscous enough to be extruded via a thin needle and, at the same time, should retain the extruded form. Such a large number of requirements completely reduced the list of candidates—different polymers and polysaccharides, to the single variant—polyvinyl alcohol (PVA). This substance is U.S. Food and Drug Administration (FDA) approved for human use, accessible in medical grade marks, dissolves in water forming fluid gels even in low concentrations, and is inert enough to mix with most usable drugs [27,28,29,30].

**Figure 4:**
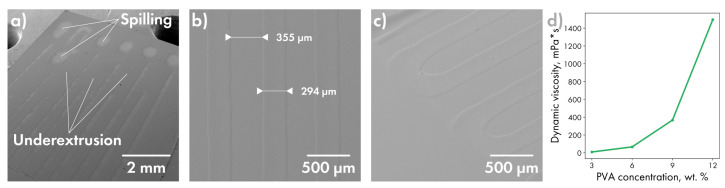
Selection of parameters for PVA gel preparation. (**a**) Print defects when using 6 wt % PVA solution; line width on a straight section (**b**) and on turns (**c**) using 9 wt % PVA solution; the dependence of the dynamic viscosity of PVA on the concentration (**d**).

The series of PVA solutions with concentrations ranging from 3 to 12 wt % was tested to obtain a minimal one with which printing is possible without the interruption of extrusion and unnecessary spilling of the gel on the substrate surface (Figure 4a).

The better performing results were achieved for the 9 wt % sample having the dynamic viscosity of about 370 mPa·s (Figure 4d). Next, the speed and flow calibration was conducted to obtain the uniform extrusion width with a minimal line broadening at the corners (Figure 4b,c) resulting in a maximum printing speed of 20 mm/s, gel flow of 0.85 mL/h and a line width of about 295 µm.

### 3.3. Proposed Idea of Release, Polymer Part

For film manufacturing polyglycolic-*co*-lactic acid (PLGA), 50:50 copolymer was chosen, because it was found to be both biocompatible and biodegradable and approved by the FDA for drug delivery, diagnostics and other medicine-related applications [31]. A low melting point of about 60 °C, good barrier properties and mechanical characteristics coupled with the ability to dissolve in non-chlorinated solvents such as acetone, tetrahydrofuran, and esters make it an excellent candidate for large-scale and environmentally friendly production [24,32,33].

To ensure safety both for the 3D printing machine and operator, it was decided to use ethyl acetate to prepare the PLGA solution; however, when we use polymer concentration of 10% and higher, which is necessary to create sufficiently thick films using the Dr. Blade technique, the gelation of the solution was observed during long-lasting experiments. To stabilize the solution, the addition of polar acetone has been proposed. Thus, to fabricate test samples, a 10 wt % PLGA solution in an ethyl acetate:acetone mixture with a ratio of 8:2 was used.

Since the final goal of our work is to obtain films suitable for the surface modification of implantable devices or dressing materials, we had to provide a film thickness sufficient for easy and safe handling. Coating parameters were optimized to obtain a base PLGA film of about 2–3 µm and a cover film of 8–10 µm thick. Such a different thickness is explained by the fact that the base film should be strong enough only to ensure defect-free removal of the film from the substrate and does not play a role in the release, since this side of the DEF is glued to the implant. The thick cover film should ensure both mechanical strength and uniform coating of the relief surface of the drug layer and play a key role in the release properties. The optimized parameters and features needed for film forming are specified in Table A1, and detailed methods are described in Section 2.2.

**Figure 5:**
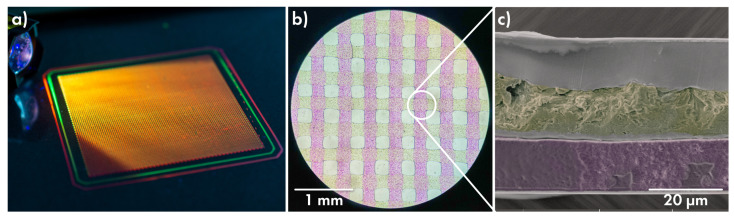
Large-area multilayered film under UV light (**a**), its optical image (**b**) and SEM image of cross-section (**c**).

To evaluate the maximum of printing capabilities, a large-area multilayered film was manufactured. Two layers of PVA matrix holding pink and yellow fluorescent colourants, separated by PLGA film, were alternately applied on the PLGA-precoated PP substrate. Figure 5 demonstrates a resulting film with an area of 36 cm^2^ (Figure 5a) and its optical image (Figure 5b). The SEM image of the cryosection (Figure 5c) in the area of line intersection makes it possible to estimate the thickness of the drug layer (about 13 µm), the lower film and separating film (roughly 2 µm each), as well as the covering film (10–15 µm).

### 3.4. Characterization of Cefazolin-Loaded Films

**Figure 6:**
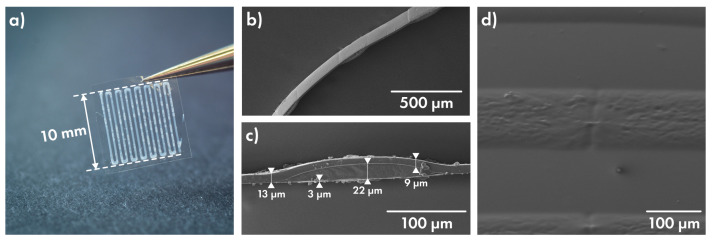
(**a**) Cefazolin-containing film sample, SEM images of its edge (**b**,**c**) and of cover film-coated drug surface (**d**).

Three sets of PLGA film samples—set **Cef100**, **Cef200** and **Cef400**—were prepared for each Cefazolin concentration in a gel matrix (100, 200 and 400 mg/mL, respectively). Each set represented a film with six gel-printed squares; the area of each sample was 1 cm^2^ (Figure 6a). Each square was cut out for a separate study of release properties. The uniformity of loading was proved for set **Cef400**; for this, nine samples were additionally printed on a base PLGA film without cover film application and then individually dissolved in 1 mL of saline. The total drug loading was calculated in terms of the sample area and amounted to 843.05 ± 34.40 µg/cm^2^. With a decrease in drug concentration in the PVA matrix, a corresponding drop in overall load is expected.

Figure 6b,c shows SEM images of the film’s edge, which were obtained via cryotom slicing for **Cef400** set. The overall film thickness in drug-filled regions is about 35 µm, and the thickness of PLGA film between tracks is about 9 µm. The cover film evenly covers the relief of the drug layer, repeating it (Figure 6d). To eliminate release contribution through the base film, all films were left on the PP substrate.

### 3.5. Release of Cefazolin from Layered Films Depending on Drug Concentration

**Figure 7:**
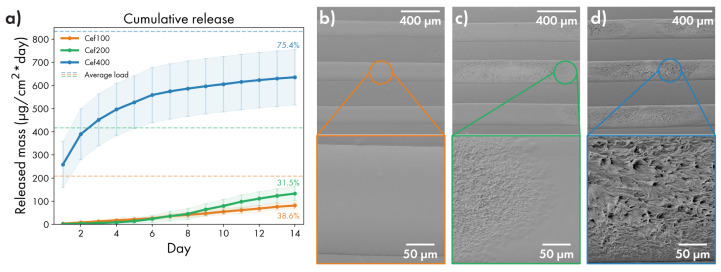
(**a**) Prolonged release profiles of Cefazolin from films prepared with various drug load (100/200/400 mg per 1 mL of PVA matrix). (**b**–**d**) SEM image for the surface of uncoated drug strips for **Cef100**, **Cef200** and **Cef400** samples, respectively.

The full scheme for the determination of kinetic of Cefazolin prolonged release is described in Section 2.4.2. Release measurements were conducted for 14 days; the resulting profiles are presented in Figure 7a. Films in groups **Cef100** and **Cef200** show similar behavior, releasing an average of 5.5 and from 5 to 13 µg/cm^2^ per day, respectively. Films with the highest Cefazolin load (group **Cef400**), in contrast, released the bulk of the loaded cargo in the first three days. Then, they gradually reduced the release speed to 7 µg/cm^2^ per day by the end of the first week.

Despite the markedly different amounts of the unsoluble component in the groups **Cef100** and **Cef200**—about 50% of PVA in **Cef100** group and 33% in **Cef200** group, a similar release in percentage terms can be seen. That fact, assuming the same barrier properties of the cover film, may point to the influence of the PVA/drug ratio not only on the amount of reagent “available” for release near the defect but also on the rate of diffusion of the dissolved drug to the defect.

The rapid release rate observable for **Cef400** samples may be indicative of a significantly increased area of defects. When comparing samples **Cef100–Cef400** without cover film (Figure 7b–d), an increase in the surface roughness due to the crystallization of Cefazolin is noticeable. For preparing **Cef100** series samples, a mixture of PVA:drug ratio near 1:1 was used, so a large amount of amorphous polymer minimized Cefazolin crystallization. With decreasing PVA content, the crystallization regions begin to be visible on **Cef200** samples and occupy the entire area of the **Cef400** sample containing about 20% of polyvinyl alcohol. Such a rough surface can lead to wetting defects, resulting in a large number of submicron pores and damage to the film under the physical impact (bending, pressure), resulting in accelerated drug elution.

Testing this hypothesis requires a series of alternative experiments, and it is beyond the focus of this study. Nevertheless, we can propose that by controlling the degree of crystallinity of the load by introducing various additives that hinder crystallization (surfactants, oligo- and polymers, etc.) or by using accelerated drying of the drug layer (printing on a heated bed, blowing with warm air), it might be possible to directly affect the degree of defectiveness of the film and thus "program" release properties in the future. In its current state, the **Cef400** series films can still be used as an effective carrier for short (up to a week) high-dose and rapid-release therapy: for example, as a dressing material or coating for temporary bone spacers [34].

**Figure 8:**
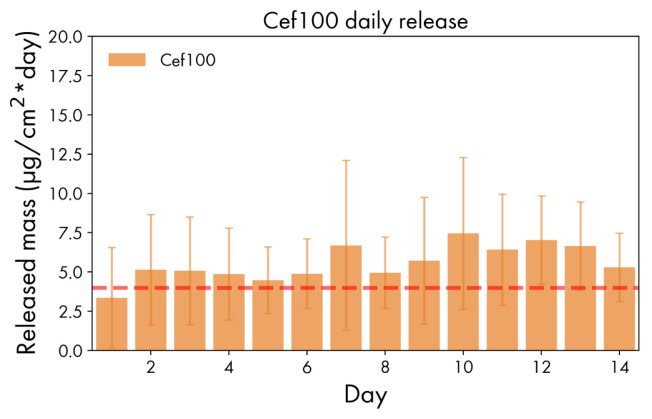
Prolonged release daily profile of **Cef100** series. Minimum Inhibitory Concentration (MIC) for MSSA is shown as a red dashed line.

Films from the **Cef100** and **Cef200** groups, on the other hand, are well suited for long-term release. Samples of **Cef100** series have excellent loading retention, slowly releasing the drug and maintaining a constant concentration in the surrounding area (Figure 8). It is worth noting that a concentration of 2–4 µg/mL is sufficient to inhibit the growth of some bacterial strains, for example, for methicillin-susceptible *Staphylococcus aureus* [35].

In order to raise the daily dosage, the increase in the content of the drug in the films can be used with no loss of release duration. Using the previously proposed tactics of introducing microdefects caused a crystallization step, or incorporating them by porogenic additives usage [36] or laser perforation methods for the cover film can significantly accelerate the rate of release of drug and will be considered in future papers.

We have demonstrated the current version of the PLACE technology. The performance of the presented method is limited by the size of the laboratory printer, the manual transition between the stages of deposition of all layers, and the residual effect of drug crystallization on release properties. A lot of further exploration on the study of the film mechanics for different polymers, tuning release properties, and methods of depositing DEFs to implants await us ahead. We demonstrated a step toward scaling production. The layer-by-layer deposition of films by doctor blading and additive technology for applying a drug layer is easily transferable to a roll-to-roll coating environment and suited for functional coating manufacturing in commercial quantities. At the same time, the ability to customize the print shape and control the dosage of the drug allows rebuilding production at any moment to modify a specific implantable device and easily work with individual implants, taking care of each patient. Obtaining a sustainable and scalable production technology is important to quickly start clinical trials and, therefore, bring the technology closer to real application.

## 4. Conclusions

In summary, we can conclude that films made by the proposed PLACE technology can be promising drug-eluting material. PLACE coatings are suitable for the functionalization of implantable medical devices with different surface areas as well as free-standing dressing patches. Considering the **Cef100** and **Cef200** series, we observed a quite uniform release of Cefazolin antibiotic in a dosage sufficient to ensure MIC in nearby tissues and keep it constant for a prolonged amount of time (2 weeks at least). The fact that the crystallization of a substance can lead to certain defects in the cover film formation requires an additional study of ways to control this phenomenon. Further study of possible methods for introducing microdefects coupled with the ability to create multilayer films with several components opens up great scope for customizing film cargo and its release properties.

## Data Availability

Not applicable.

## Supplementary Materials

Circuit diagrams, 3D models and Arduino sketches can be found at https://github.com/Pavel8Pro/PLACE_pump_SM.

## Abbreviations

The following abbreviations are used in this manuscript:

AG	Applicator gap
CS	Coating speed
DEF	Drug-eluting film
DI	Deionized (water)
MIC	Minimum Inhibitory Concentration
MSSA	Methicillin-susceptible *Staphylococcus aureus*
PDMS	Polydimethylsiloxane
PGA	Polyglycolic acid
PLACE	Printed Layered Adjustable Cargo Encapsulation
PLGA	Poly(lactic-*co*-glycolic acid)
PP	Polypropylene
PLA	Polylactic acid
PCL	Polycaprolactone
ZO	Z-offset

## Appendix A

**Table A1:** Optimized parameters and features needed to film forming.

Manufacture Stage	Optimized Manufacture Parameters	Resulted Features
PLGA solution	Solvent	Acetone: Ethyl acetate = 2:8
	Concentration	10 wt %
Base film	CS ^1^ = 20 mm/s; AG ^2^ = 100 µm	Film thickness = 3 µm
Cover film	CS = 20 mm/s; AG = 400 µm	Film thickness = 12 µm
Drug layer	Needle size 23G;	Line width = 295 µm
	Flow = 0.85 mL/h	Line spacing = 335 µm
	Printing speed = 15 mm/s	Line max. height ^3^ = 11 µm
	PVA matrix conc. = 9 wt %	Matrix viscosity = 400 mPa·s

^1^ Coating Speed. ^2^ Applicator Gap. ^3^ For pure PVA matrix.
